# Linear vs. Nonlinear Extreme Learning Machine for Spectral-Spatial Classification of Hyperspectral Images

**DOI:** 10.3390/s17112603

**Published:** 2017-11-13

**Authors:** Faxian Cao, Zhijing Yang, Jinchang Ren, Mengying Jiang, Wing-Kuen Ling

**Affiliations:** 1 School of Information Engineering, Guangdong University of Technology, Guangzhou 510006, China; faxiancao@foxmail.com (F.C.); mengyingjianggdut@foxmail.com (M.J.); yongquanling@gdut.edu.cn (W.-K.L.); 2 Department of Electronic and Electrical Engineering, University of Strathclyde, Glasgow G1 1XW, UK; jinchang.ren@strath.ac.uk

**Keywords:** hyperspectral image (HSI), extreme learning machine (ELM), spectral-spatial classification, discriminative random field (DRF), loopy belief propagation (LBP)

## Abstract

As a new machine learning approach, the extreme learning machine (ELM) has received much attention due to its good performance. However, when directly applied to hyperspectral image (HSI) classification, the recognition rate is low. This is because ELM does not use spatial information, which is very important for HSI classification. In view of this, this paper proposes a new framework for the spectral-spatial classification of HSI by combining ELM with loopy belief propagation (LBP). The original ELM is linear, and the nonlinear ELMs (or Kernel ELMs) are an improvement of linear ELM (LELM). However, based on lots of experiments and much analysis, it is found that the LELM is a better choice than nonlinear ELM for the spectral-spatial classification of HSI. Furthermore, we exploit the marginal probability distribution that uses the whole information in the HSI and learns such a distribution using the LBP. The proposed method not only maintains the fast speed of ELM, but also greatly improves the accuracy of classification. The experimental results in the well-known HSI data sets, Indian Pines, and Pavia University, demonstrate the good performance of the proposed method.

## 1. Introduction

The main goal of HSI classification is to assign each pixel of the hypercube into a different class according to the spectral and spatial characteristics [1]. Since each pixel of HSI has many spectral features, it is difficult to classify HSI with limited samples, due to the curse of dimensionality. There are some typical algorithms for HSI image classification, such as the support vector machine (SVM) [2] and sparse multinomial logistic regression (SMLR) [3]. Many techniques have been proposed for feature extraction and dimensionality reduction [4,5], such as singular spectrum analysis (SSA) [6,7,8,9], principal component analysis (PCA) [10,11], and spectral-spatial classification methods [12]. However, there are still many challenges facing HSI classification, for example, the data structure of each pixel in the HSI data is actually a vector, corresponding to the responses from different spectral bands. The dimension of the vector equals the number of spectral bands, usually in the scale of hundreds or even thousands. As a result, it is still a challenging problem for the efficient and effective classification of HSI, especially with limited training samples.

As a new machine learning approach that has a single-hidden layer feedforward neural network, ELM has received much attention due to its good performance. It has been proven to be a promising algorithm in pattern recognition fields [13,14,15,16,17]. Compared to SVM and other state-of-the-art algorithms, ELM has the following advantages [17]: a very simple structure, higher generalization, and high computational efficiency without tuning additional parameters. The original ELM is a linear operation, so we call it linear ELM (LELM). Although it has the above advantages, the classification accuracy is not very high when applied to hyperspectral images. Kernel ELM (KELM) [18] and sparse ELM [19] are improvements of LELM and achieve better classification results than LELM. The classification accuracy of KELM is improved but still not high enough when applied to HSI. The main reason that LELM and KELM cannot achieve a high accuracy of classification is that they only make use of the spectral information of HSI, without considering the spatial information of HSI. As spatial information reflects the local property of HSI data sets, it is very important for the classification of HSI.

In order to improve the classification results of HSI, some spectral-spatial classification methods based on ELM have been proposed. For example, Zhou et al. [20] combined ELM with composite kernels (ELM-CKs) for HSI classification. In [21], a Gabor filter with ELM (G-ELM) was proposed for spectral-spatial HSI classification. In [22], a superpixel was proposed for spectra-spatial feature extraction followed by ELM for classification. In [23], extended morphological profiles with ELM (EMP-ELM) were introduced for HSI classification. Although these ELM-based spectral-spatial methods have produced reasonably good results, their performance can be further improved by using more effective spatial features, as discussed below.

To further improve the performance of the ELM-based spectral-spatial classification of HSI, a loopy belief propagation (LBP) algorithm is used [24,25]. As a conditional probability model, LBP can be considered as a generalization of the Markov chain and can effectively describe the correlation of all the nodes/pixels in the field. It is based on the Markov random field (MRF) which assumes that the neighboring pixels likely belong to the same class [26,27,28]. The principle of LBP for classification is to calculate the marginal probability based on the characteristics of the samples. As an extension to ELM, KELM has been taken as an improved solution to combine MRF for the better classification of HSI [29]. However, based on comprehensive experiments and analysis, we found that the linear ELM is a better choice than KELM for the spectral-spatial classification of HSI.

LELM is a type of linear operation, so its final mapping results will not change the characteristics of pixels in HSI. Nevertheless, KELM is a type of nonlinear operation, namely NLELM, and its final mapping results will disturb the features of pixels in the same class. If we use the output of NLELM as the input for MRF or LBP, the structure of NLELM will seriously disturb the original information of HSI. Then, it cannot fully utilize the spectral information and spatial information of HSI and will degrade the classification accuracy. For example, the NLELM and MRF are combined for the classification of HSI in [29], called NLELM-MRF. Since NLELM disturbs the features of the pixels in the same class, it causes the classification accuracy to be relatively low [29]. To this end, LELM is used here with LBP for the spectral-spatial classification of HSI to achieve a higher classification accuracy.

As mentioned above, the LBP algorithm is based on the MRF, which uses the information of the node to transmit information and update the current MRF state [3]. It is a kind of approximate calculation based on MRF. This algorithm is an iterative method, which can solve the problem of probabilistic inference in probabilistic graphical models. After many iterations of probability, the belief of all the nodes is no longer changed. Then, the LBP algorithm can converge to its optimal solution. Since the pixels of HSI that need to be classified are just a part of HSI, it means that not all the pixels in HSI need to be classified. If we use LBP to classify HSI directly, it may cause ill-posed problems. In view of this, we make some improvement of LBP for HSI classification. The pixels of background of HSI are ignored in the process of LBP. The proposed framework will fully make use of the spectral and spatial information by ELM to improve the classification accuracy dramatically. Experimental results demonstrate the better performances compared with other state-of-the-art methods in the same situation.

The remainder of this paper is divided into the following sections: Section 2 describes the experimental data and the details of the proposed method. Section 3 shows the extensive experimental results and discussions. The conclusions are summarized in Section 4.

## 2. Materials and Methods

In this section, we first introduce the experimental data sets, and then we elaborate the proposed method based on LELM and LBP.

### 2.1. HSI Data Set

The experimental data sets include two well-known HSI datasets, which are detailed below.

(1) Indian Pines: The Indian Pines HSI data set [3] is based on the urban image collected in June 1992 by the AVIRIS sensors over the Indian Pines region in North-western Indiana. The Indian Pines scene contains two-thirds agriculture and one-third forest or other perennial vegetation. There are two major dual lane highways and a rail line, as well as some low density housing, other built structures, and smaller roads. Since the scene is taken in June, some of the crops present, including corn and soybeans, are in the early stages of growth with less than 5% coverage. The data set has 145 × 145 pixels, each of which has 200 spectral bands after removing 20 water absorption bands ranging from 0.2 to 2.4 μm. There are 16 classes in total (e.g., corns, soybeans and, wheat), with 10,366 pixels that need to be classified. This data set can be downloaded at http://www.lx.it.pt/~jun/.

(2) Pavia University: The Pavia university HSI data set [1] was acquired in 2001 by the Reflective Optics System Imaging Spectrometer (ROSIS), flown over the city of Pavia, Italy. The sensor collected an HSI data set in 115 spectral bands ranging from 0.43 to 0.86 μm with a spatial resolution of 1.3 m/pixel. A total of 103 bands were selected for experiments after removing 12 noisiest bands. The image scene contains 610 × 340 pixels and there are nine classes in total, with 42,776 pixels that need to be classified. This data set can be downloaded at http://www.lx.it.pt/~jun/.

### 2.2. Normalization

Normalization is a preprocessing step for HSI classification. As an important preprocessing step for HSI classification, a number of normalization approaches have been proposed. For simplicity and consistency, we chose the Max method for normalization as it is a widely used method [30]. Let $X=(X_{1},{\;X}_{2},\ldots \;,{\;X}_{N})\in R^{N\times d}$ be the HSI data, which has *N* samples and each sample has *d* features. The Max method divides the maximum value of the whole data set which can be expressed as:

(1) $$x_{ij}=X_{ij}/\max(X)$$

where $X_{ij}$ is any pixel value of the HSI data, and max() is the largest value of all the data in the HSI.

### 2.3. Linear ELM

Let $x=(x_{1},{\;x}_{2},\ldots \;,{\;x}_{N})\in R^{N\times d}$ be the HSI data after normalization, where $y=\left(y_{1},{\;y}_{2},\;\ldots ,{\;y}_{N}\right)\in R^{N\times M}$ denotes the class labels. As a new learning algorithm, ELM [17] is a single layer feedforward neural network, which can be modeled as:

(2) $$\overset{L}{\underset{j=1}{\sum }}\beta_{j}G(w_{j}^{T}x_{i}+b_{j})=y_{i}$$

where $w_{i}={\left(w_{i1},\;w_{i2},\;\ldots ,\;w_{iL}\right)}^{T}$ is the weight vector connecting the input layer with the hidden layer of the *i*-th sample; $b_{j}$ is the bias connecting the input layer with the hidden layer of the *i*-th sample and $\beta_{j}$ is the output weight vector of the *i*-th sample; *T* is the transpose operation; and *g*() is the activation function of the hidden layer. The main steps of classification with ELM are as follows:

**Step1:** Assign random input $w_{i}$ and bias $b_{i}$, i=1, 2, …, N for the input layer.

**Step2:** Calculate the output matrix of hidden layer *G* as:

(3) $$G\left(w_{1},w_{2},\ldots ,w_{N};x_{1},x_{2},\ldots ,\;x_{N};b_{1},b_{2},\ldots ,b_{N}\right)=\left[\begin{array}{ccc} g_{11}\left(w_{11}x_{11}+b_{11}\right) & \ldots & g_{1L}\left(w_{1L}x_{1L}+b_{1L}\right)\\ \ldots & \ldots & \ldots \\ g_{N1}\left(w_{N1}x_{N1}+b_{N1}\right) & \ldots & g_{NL}\left(w_{NL}x_{NL}+b_{NL}\right) \end{array}\right]$$

**Step3:** Calculate the output matrix β:

(4) $$\beta =G^{†}y$$

where $\beta ={\left[\beta_{1},\ldots ,\;\beta_{L}\right]}_{L\times M}^{T}\;$ and † is the Moore-Penrose generalized inverse of the hidden layer matrix.

**Step4:** The result of the final classification of ELM can be expressed by the following equation:

(5) f(x)=G∗β

The execution time of ELM can be greatly reduced because the input weight and bias of ELM are randomly generated, and the output weight can be directly computed as $\beta =G^{†}*y$. Any piecewise continual function can be used as the hidden layer activation function. Obviously, ELM is a lineal operation.

### 2.4. Nonlinear ELM

The classification problem for NLELM [22] can be formulated as:

(6) $$Minimize:L_{NLELM}=\frac{1}{2}∥\beta {∥}_{F}^{2}+C\frac{1}{2}\overset{N}{\underset{i=1}{\sum }}∥\varepsilon_{i}{∥}_{2}^{2}\;subject\;to:\;h\left(x_{i}\right)\beta =t_{i}^{T}-\varepsilon_{i}^{T},\;i=1,\;\ldots ,\;N$$

where $\varepsilon_{i}=\left[\varepsilon_{i,1},\ldots \;,\varepsilon_{i,M}\right]\;$ is the error vector of the *M* output nodes relative to the sample $x_{i}$. $h\left(x_{i}\right)$ is the output of the *i*-th sample between the hidden layer and the input layer. Based on the KKT theorem, Equation (6) is equivalent to solve the following dual optimization problem:

(7) $$L_{NLELM}=\frac{1}{2}∥\beta {∥}_{F}^{2}+C\frac{1}{2}\overset{N}{\underset{i=1}{\sum }}∥\varepsilon_{i}{∥}_{2}^{2}-\overset{N}{\underset{i=1}{\sum }}\sum_{j=1}^{M}\alpha_{i,j}\left(h\left(x_{i}\right)\beta_{j}-t_{i,j}^{T}+\varepsilon_{i,j}^{T}\right)$$

where $\beta_{j}$ is the vector of weight between the hidden layer and output layer. $\alpha_{i,j}$ is the Lagrange multiplier. Based on the KKT theorem, we can derive that:

(8) $$\frac{\partial L_{NLELM}}{\partial \beta_{j}}=0\to \beta =H^{T}\alpha$$

(9) $$\frac{\partial L_{NLELM}}{\partial \varepsilon_{i}}=0\to \alpha_{i}=C\varepsilon_{i}$$

(10) $$\frac{\partial L_{NLELM}}{\partial \alpha_{i}}=0\to h\left(x_{i}\right)\beta_{j}-t_{i}^{T}+\varepsilon_{i}^{T}$$

where *i* = 1, …, *N*, $\alpha_{i}={\left[\alpha_{i,1},\alpha_{i,2},\;\ldots \;,\alpha_{i,M}\right]}^{T}$ and $\alpha ={\left[\alpha_{1},\alpha_{2},\ldots \;,\alpha_{N}\right]}^{T}$. Now, the output weight β can be formulated as:

(11) $$\beta =(\frac{I}{C}+H^{T}H{)}^{-1}H^{T}y.$$

The hidden neurons are unknown. Any kernel satisfying the Mercer’s conditions can be used:

(12) $$\Omega_{KELM}=HH^{T}:\Omega_{KELM}\left(x_{i},x_{j}\right)h\left(x_{i}\right)h{\left(x_{j}\right)}^{T}=K\left(x_{i},x_{j}\right)$$

In general, the Gaussian kernel is chosen:

(13) $$K_{NLELM}\left(x_{i},x_{j}\right)=\exp\left(-\frac{∥x_{i}-x_{j}{∥}^{2}}{2*\sigma_{NLELM}}\right)$$

Then, the NLELM can be constructed using the kernel function.

Although NLELM can achieve a higher classification accuracy than LELM if we just consider the spectral information, it may degrade the performance in the spectral-spatial classification of HSI. As a result, we will choose the LELM with LBP for the spectral-spatial classification of HSI, yet the performance of LELM and NLELM will be compared in the experiments.

### 2.5. Using LBP Based Spatial Information to Improve the Classification Accuracy

To further extract the spatial information, the output of LELM is used as the input of LBP. The posterior density p(y/x) is obtained according to the feature x, which is the output of LELM. We adopt the discriminative random field (DRF) [26] as:

(14) $$P\left(y/x\right)=\frac{1}{Z\left(x\right)}\exp(\sum^{\text{​}}\log p(y_{i}/x_{i})+\sum^{\text{​}}\log p\left(y_{i},y_{j}\right))$$

where Z(x) is the partition function. The term $\log p(y_{i}/x_{i})$ is the association potential that models the likelihood of label $y_{i}$ given the feature $x_{i}$, and $\log p\left(y_{i},y_{j}\right)$ is the interaction potential.

We adopt an isotropic MLL prior to the model image of class label y in order to use the spatial information of HSI. This prior belongs to the MRF class and encourages piecewise smooth segmentations. It tends to produce solutions where the adjacent pixels are likely to belong to the same class [3]. The MLL prior has been widely used in image segmentation problems [31,32,33,34] and is a generalization of the Ising model [35,36,37]. It can be formulated as:

(15) $$p\left(y\right)=\frac{1}{Z}exp^{\mu \sum^{\text{​}}\delta \left(y_{i},y_{j}\right)}$$

where μ is a tunable parameter controlling the degree of smoothness, *Z* is a normalization constant for the density, and δ(y) is the unit impulse function. The pairwise interaction term $\delta \left(y_{i},y_{j}\right)$ assigns a high probability to the neighborhoods. The setting of the smoothness parameter, *μ*, will be discussed in Section 3.2.

A maximum a posteriori (MAP) estimate will minimize the Bayesian risk associated with the zero-one loss function [3]. The MAP estimate of y can be given by:

(16) y^=argminy∑​−log(yi/xi)−μ∑​δ(yi−yj)

This is a combinatorial optimization problem having pairwise interaction terms. An alternative MAP solution is the MAP marginal (MAM) solution, which minimizes the Bayesian risk associated with the zero-one loss function. The MAM estimation of label $y_{i}$ can be formulated as:

(17) yi^=argmaxyiq(yi/x)

where $q(y_{i}/x)$ is the marginal density of p(y/x) with respect to $y_{i}$. The computation of the marginal density of p(y/x) in (14) is difficult [3]. Since the LBP is an efficient approach to estimate Bayesian beliefs [24] in graphical model, here we will use LBP to estimate the MAM solution and let the output of LELM $y_{LELM}^{*}$ be the input of LBP.

**Figure 1:**
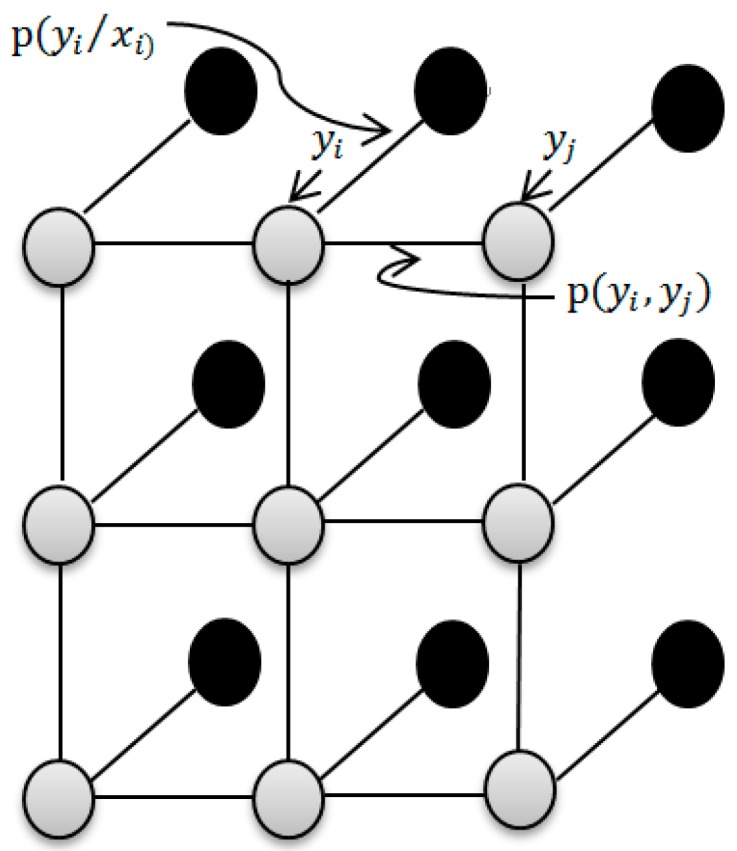
Graph example of MRF.

Figure 1 is a graphical example of MRF, where each node represents a random variable or a hidden node, and the class label $y_{i}$ is associated with each input feature $x_{i}$. In the graphical example of MRF,${\;\psi }_{\mathrm{ij}}\left(y_{i},y_{j}\right)=p\left(y_{i},y_{j}\right)$ denotes the interaction potential that penalizes the dissimilar pair of neighboring labels. $\varphi_{i}\left(y_{i},x_{i}\right)=p\left(y_{i}/x_{i}\right)$ stands for the association potential of label $y_{i}$ with respect to evidence. Suppose we observe some information about $x_{i}$. Each node has the state value $y_{i}$, and the observation value $x_{i}$. $\varphi_{i}\left(y_{i},x_{i}\right)$ reflects the existence of statistical dependence. $\psi_{\mathrm{ij}}\left(y_{i},y_{j}\right)$ is the potential energy between adjacent neighbor nodes, and reflects the compatibility between the node variables $y_{i}$ and $y_{j}$.

**Figure 2:**
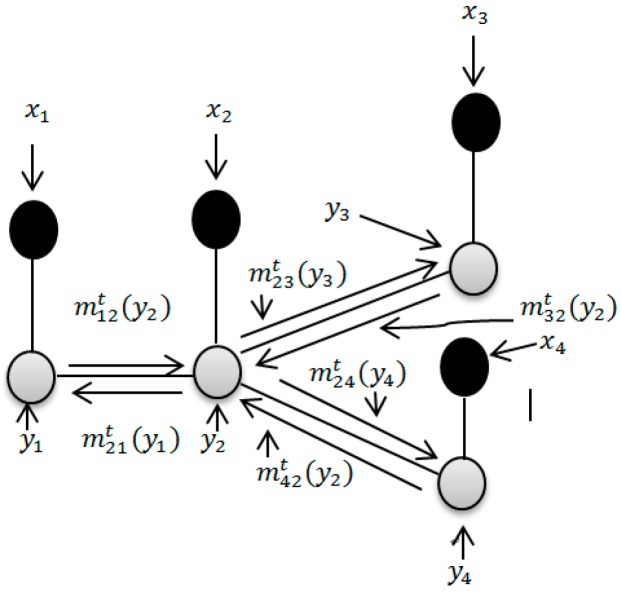
Message passing of LBP at *t*-th iteration.

Figure 2 provides a graphical example of an undirected network. Since LBP is an iterative algorithm, at the *t*-th iteration, the message sent from node *i* to its neighbor node j∈N(i) can be given by the following equation:

(18) $$m_{\mathrm{ij}}^{t}\left(y_{j}\right)=\frac{1}{Z}\underset{y_{i}}{\sum }\psi \left(y_{i},y_{j}\right)\varphi \left(y_{i},x_{i}\right)\underset{k\in N\left(i\right)\left\{j\right\}}{\prod }m_{\mathrm{ki}}^{t-1}\left(y_{i}\right)$$

where *Z* is a normalization constant.

Assume that $b_{i}^{t}\left(y_{i}\right)$ is the belief of node *i* at the *t*-th iteration, it can be represented by the following equation:

(19) $$b_{i}^{t}\left(y_{i}=k\right)=q\left(y_{i}=k/x\right)=\;\varphi \left(y_{i}=k\right)\underset{j\in N\left(i\right)}{\prod }m_{\mathrm{ji}}^{t}\left(y_{i}=k\right)$$

Finally, we can estimate the final solution by maximizing the posterior marginal for node *i*:

(20) yi^=argmaxyiq(yi/x)=argmaxyibit(yi)

As we know, not all the pixels, but only a part of the HSI needs to be classified. For instance, the size of the HSI data set of Indian Pines is 145 × 145 × 200, so the size of ground-truth is 145 × 145. But only 10,366 out of 21,025 pixels need to be classified. It may cause ill-posed problems if we use LBP directly with all the pixels. In view of this, we make some improvement of LBP (ILBP) in order to solve this problem, where we discard the pixel that belongs to the background, i.e., we just consider the pixels that need to be classified. The proposed method is summarized in Algorithm 1.
**Algorithm 1** Spectral-Spatial Classification for HSI Based on LELM and ILBP**Input X:** the HSI image; X1: training samples; X2: test samples; Y1: The desired output of training sample; *L*: number of hidden node of ELM; *g()*: activation function of hidden layer of ELM. (1) **Normalization:** Let $X1^{*}=X1/\max(X)$, $X2^{*}=X2/\max(X)$. (2) **LELM training:**
  Step 1: Randomly generate the input weights, $w_{i}$, and bias, $b_{i}$.   Step 2: Calculate the hidden layer of the output matrix: $$G1=g\left(w_{i}^{T}*X_{1}^{*}+b_{i}\right)$$   Step3: Calculate the output weight: $$\beta =G^{†}*Y1$$**Output of LELM:** Calculate the hidden layer matrix of the test samples: $G2=g\left(w_{i}^{T}*X2^{*}+b_{i}\right)$.          Obtain the output result of LELM: $Y_{ELM}=G2*\beta$. (3) **Spatial Classification by ILBP:**Step1: Find the index of adjacent pixels of training samples and test samples and eliminate the pixels of the background.Step2: Calculate the marginal of MPA as follows:For *t* = 1: time of iterations For *j* = 1: number of pixels  If *j* ~= test samples   Don’t calculate the marginal of MAM.   Or   Calculate the marginal of MAM: $$m_{ij}^{t}\left(y_{j}\right)=\frac{1}{Z}\underset{y_{i}}{\sum }\psi \left(y_{i},y_{j}\right)\phi \left(y_{i},x_{i}\right)\underset{k\in N\left(i\right)\left\{j\right\}}{\prod }m_{ki}^{t-1}\left(y_{i}\right)$$  Then the belief of node *i* at the *t*-th iteration can be represented as: $$b_{i}^{t}\left(y_{i}=k\right)=q\left(y_{i}=k/x\right)=\;\phi \left(y_{i}=k\right)\underset{j\in N\left(i\right)}{\prod }m_{\mathrm{ji}}^{t}\left(y_{i}=k\right)$$   End  End The final solution for node i can be obtained by maximizing the posterior marginal: yi^=argmaxyiq(yi/x)=argmaxyibit(yi).

## 3. Results and Discussions

In this section, the proposed method will be evaluated and relevant results are discussed in details. The experimental datasets include two well-known HSI datasets, i.e., Indian Pines and Pavia University.

### 3.1. Parameter Settings

All the experimental results are assessed by the overall accuracy (OA), average accuracy (AA), and kappa statistics (k) [35]. In order to avoid the effects induced by the selection of training samples, ten independent Monte Karlo runs are performed and OA, AA, and k are all averaged by ten runs.

In order to compare the performance of the proposed method with other classifiers, we show the parameter settings used in the experiments. The parameters of SMLR and KSMLR are the same as suggested in [38] (noting that the SMLR and KSMLR are implemented via variable splitting and augmented Lagrangian (LORSAL) [39], which can decrease the computation time of SMLR and KSMLR). The cost function $C=2^{b}$ of NLELM is in the range of b=[0, 1, 2,… , 10], the kernel function in (12) is used as the Gaussian RBF with $\sigma_{NLELM}=2^{\tau }$, τ = {−9, −8, …, 0,…8, 9}, and the parameters are set as b = 9, τ = −1. The parameters of NLELM are set by choosing the best in our experiments. For LELM, hidden node *L* in (3) is a very important parameter and we will evaluate the impact in the next subsection. The parameter μ in (15) is a tunable parameter controlling the degree of smoothness, which is set to μ=20 for Indian Pines and Pavia University. We will further evaluate the impact on the proposed approach in the next subsection. Note that the output of LELM and NLELM represent the probability output. All the experiments are conducted in MATLAB R2016b on a computer with 3.50 GHz CPU and 32.0 G RAM.

### 3.2. Impact of Parameters L and μ

In this subsection, we will evaluate the impact of the hidden neurons of LELM, *L*, and the smoothness parameter, μ, using the Indian Pines and Pavia University datasets. Table 1 displays the number of training samples and test samples.

**Figure 3:**
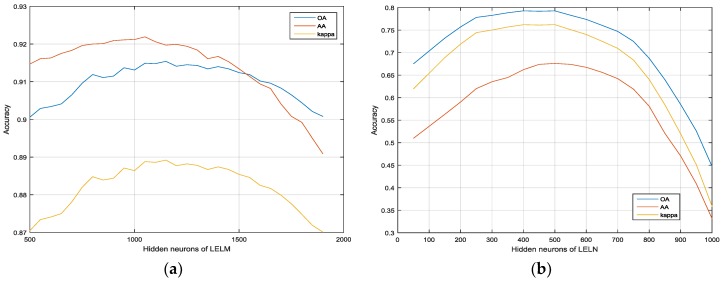
The impact of hidden neurons of ELM in the datasets: (**a**) Indian Pines; (**b**) Pavia University.

Figure 3 shows the OA, AA, and kappa statistic results as a function of variable *L* with the training samples of 1043 and 3921 in the Indian Pines and Pavia University, respectively (about 9% and 10% of the total samples, respectively). The training samples are randomly selected from each class in each Monte Carlo Run. From Figure 3a,b, we can see that the classification accuracies of LELM indeed depend on the hidden neurons, so we should choose the best hidden neurons for LELM in order to improve the classification performance in the sequential spatial information classification. We can see that the best hidden neurons value of LELM for Indian Pines is about 450 and the best hidden neurons value of LELM for Pavia University is about 1050. Therefore, we will set the hidden neurons values as 450 for Indian Pines and 1050 for Pavia University.

**Figure 4:**
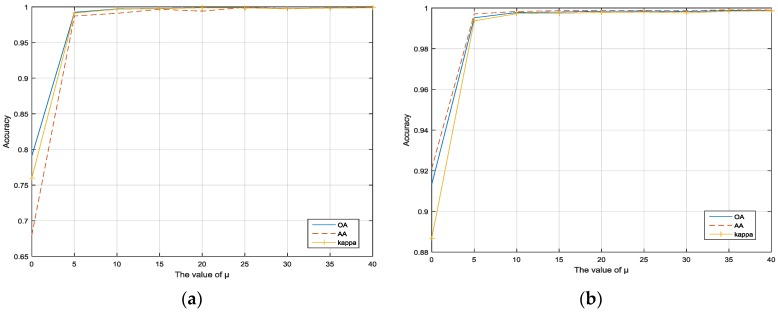
The impact of sparseness parameters μ in the datasets: (**a**) Indian Pines; (**b**) Pavia University.

Figure 4a,b show the OA, AA, and kappa statistic as a function of variable μ where the values of μ range from {0, 5, …, 40} in the experiments. It can be seen that the performance of the proposed framework depends on the smoothness parameter, μ. However, the classification performance maintains a high accuracy as  μ  is increasing and it tends to be almost unchanged when μ≥20. So in the experiments, it is set as μ=20 for Indian Pines and Pavia University. This also demonstrates that the proposed framework is very robust.

### 3.3. The Experiment Resutls and Analysis

In this subsection, we will evaluate the HSI classification accuracy of the proposed method in the two HSI datasets by comparing it with other state-of-the-art methods, including the sparse multinomial logistic regression (SMLR), kernel sparse multinomial logistic regression (KSMLR) [3], nonlinear ELM (NLELM), linear ELM (LELM) [13], SMLR-LBP, KSMLR-LBP, and NLELM-LBP. It is worth noting that the SMLR, KSMLR, LELM, and NLELM are spectral classification methods, i.e., pixel-based, while the SMLR-LBP, KSMLR-LBP, LELM-LBP, and NLELM-LBP are spectral-spatial classification methods. For the normalization, we use the Max method as in Equation (1) for all the algorithms. Table 1 shows the numbers of training samples and testing samples of Indian Pines and Pavia University.

**Figure 5:**
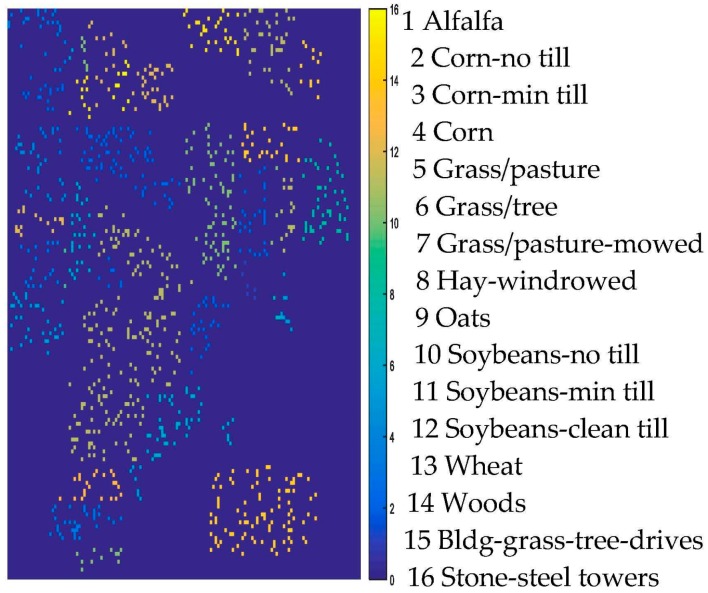
AVIRIS Indian Pines training maps.

**Figure 6:**
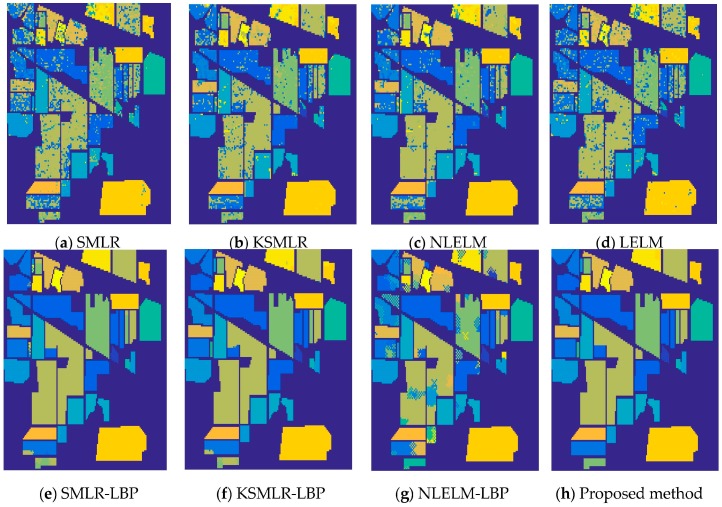
The overall accuracy of Indian Pines image: (**a**) SMLR (OA = 75.76%); (**b**) KSMLR (OA = 84.34%); (**c**) NLELM (OA = 86.93%); (**d**) LELM (OA = 79.43%); (**e**) SMLR-LBP (OA = 98.26%); (**f**) KSMLR-LBP (OA = 99.05%); (**g**) NLELM-LBP (OA = 87.95%); (**h**) Proposed method (OA = 99.75%).

For an illustration, Figure 5 shows the training samples of the Indian Pines data. Figure 6a–h show the classification results obtained by different methods for the Indian Pines data. Moreover, Table 2 shows all the comparable results of different classifiers. From Table 2, it is obvious that the classifiers with spatial information (the proposed method, NLELM-LBP, SMLR-LBP, KSMLR-LBP) show a clear advantage over their pixel-only counterpart. NLELM obtains the best pixel-only classification results, but the results of NLELM-LBP are not good. This validates that the nonlinear transform will disturb the original salient feature of the original pixels. The reason of the bad results of SMLR is due to the fact that SMLR needs to iterate and the outputs of SMLR will also disturb the original salient feature of the pixels. KSMLR-LBP achieves a slightly higher result than SMLR-LBP.

The kernel operation is better than the non-kernel operation with the pixel-only classifier. Nevertheless, the result of KSMLR-LBP is still lower than the proposed method. Our proposed spectral-spatial method based on LELM and ILBP achieves the best recognition results, when compared with LELM, NLELM, SMLR, KSMLR, NLELM-LBP, SMLR-LBP, and KSMLR-LBP. This is due to the usage of the linear transform to keep the original salient features of pixel, and the ILBP to extract the spatial features.

**Figure 7:**
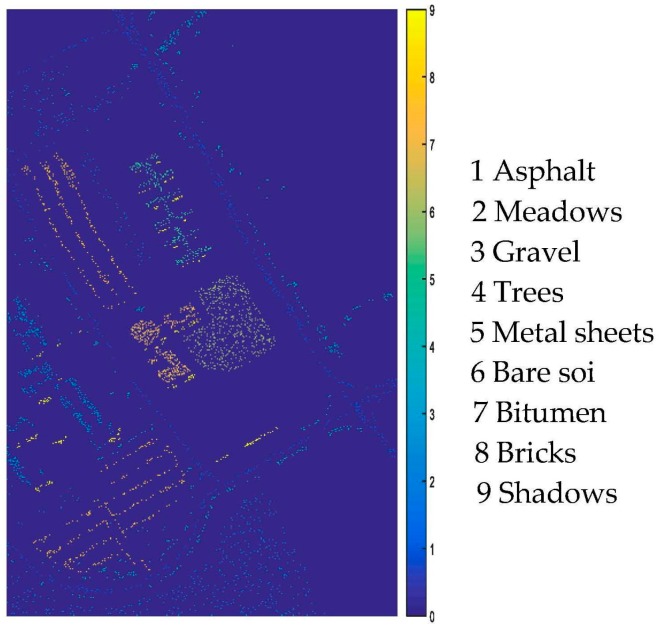
Pavia University training maps.

**Figure 8:**
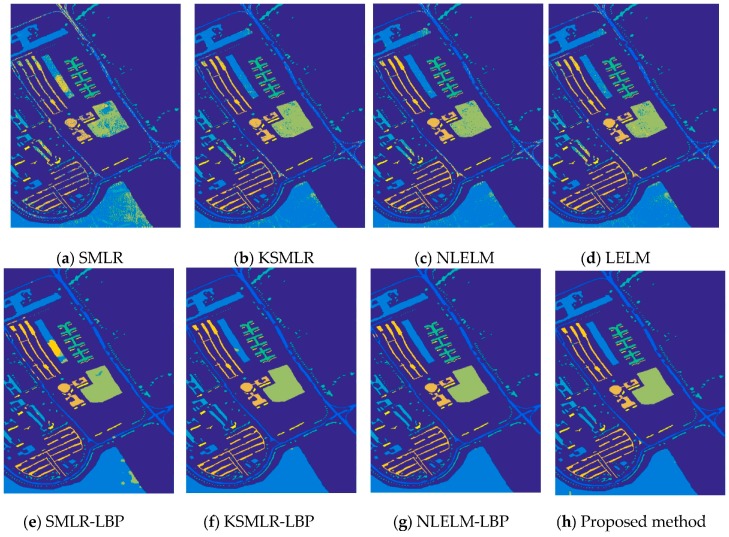
The overall accuracy of Pavia University image: (**a**) SMLR (OA = 78.78%); (**b**) KSMLR (OA = 93.00%); (**c**) NLELM (OA = 93.94%); (**d**) LELM (OA = 91.23%); (**e**) SMLR-LBP (OA = 95.68%); (**f**) KSMLR-LBP (OA = 99.42%); (**g**) NLELM-LBP (OA = 99.61%); (**h**) Proposed method (OA = 99.82%).

Figure 7 shows the training samples of Pavia University, and Figure 8 shows the classification results of Pavia University and the classification details are reported in Table 3. It can be seen that the proposed framework also achieves the highest accuracy among all the methods.

In the last line of Table 2 and Table 3, we report the average computation time of all the methods for the Indian Pines with 1043 training samples and Pavia University with 3921 training samples. We test for ten Monte Carlo runs, respectively. It is obvious and reasonable that the classifiers with spectral-spatial information cost more time than the pixel-only counterpart. From the last line of Table 2, we can also see that the proposed method has a very similar computation time as SMLR-LBP for Indian Pines. However, the proposed method achieves a higher classification accuracy than SMLR-LBP. The proposed method achieves a higher classification accuracy than NLELM-LBP and KSMLR-LBP with much less computation time. From the last line of Table 3, we can get the same conclusion for the Pavia University database. To sum up, the proposed method has achieved a higher accuracy than KSMLR-LBP, NLELM-LBP with much less computation time. It is obvious that the proposed LELM-LBP maintains the salient features of HSI very well, so it can obtain a higher accuracy than other spectral-spatial methods with a high computational efficiency.

### 3.4. The Experiment Resutls and Analysis

In this subsection, we compare the proposed approach with other spectral-spatial ELM-based methods. The classification results are shown in Table 4. The classification accuracies of EMP-ELM, S-ELM, and G-ELM are directly taken from [21,22,23], respectively. From Table 4, we can see that the proposed method achieves the best classification accuracies among all these four methods.

## 4. Conclusions

In this work, we proposed a new framework for HSI classification using spectral-spatial information with LELM and LBP. The LELM method is used to learn a spectral classifier for the original HSI data and keep the salient features of HSI. The spatial information is modeled based on LBP in order to improve the classification accuracy of HSI. The proposed method maintains the salient feature of HSI for the spatial-based classification. Experimental results show the superiority of the proposed method.

In future work, we will focus on learning the dictionary of each class in the spectral domain for LELM in order to further improve the classification of LELM. In order to improve the classification results further, we will resort to Spatial Filtering [40]. Moreover, we will also decrease the time-consuming issue by resorting to the extended multi-attribute profiles (EMAPs) [41] method.

## Figures and Tables

**Table 1:** The training sample and test samples of Indian Pines and Pavia University.

Indian Pines						Pavia University		
Class	Train	Test	Class	Train	Test	Class	Train	Test
Alfalfa	6	54	Oats	2	20	Asphalt	548	6631
Corn-no till	144	1434	Soybeans-no till	97	968	Meadows	548	18,649
Corn-min till	84	834	Soybeans-min till	247	2468	Gravel	392	2099
Corn	24	234	Soybeans-clean till	62	614	Trees	524	3064
Grass/pasture	50	497	Wheat	22	212	Metal sheets	265	1345
Grass/tree	75	747	Woods	130	1294	Bare soil	532	5029
Grass/pasture-mowed	3	26	Bldg-grass-tree-drives	38	380	Bitumen	375	1330
Hay-windrowed	49	489	Stone-steel towers	10	95	Bricks	514	3682
Total				1043	10366	Shadows	231	947
						Total	3921	42,776

**Table 2:** Indian Pines: overall, average, and individual class accuracy (in percent) and k statistic of different classification methods with 10% training samples. The best accuracy in each row is shown in bold.

Class	SMLR	KSMLR	LELM	NLELM	SMLR-LBP	KSMLR-LBP	NLELM-LBP	PROPOSED METHOD
Alfalfa	30.52	74.26	35.37	71.11	97.78	100	90.37	**100.00**
Corn-no till	75.87	82.49	79.27	85.82	99.02	99.40	85.68	**99.68**
Corn-min till	51.35	70.86	58.26	72.58	92.55	97.35	68.79	**99.22**
Corn	37.35	68.68	43.29	69.10	99.27	95.00	77.44	**100.00**
Grass/pasture	86.82	89.46	89.76	93.64	97.36	98.23	93.64	**99.28**
Grass/tree	94.28	96.37	96.32	97.39	**100.00**	**100.00**	95.70	**100.00**
Grass/pasture-mowed	6.92	45.00	11.54	70.38	71.92	91.54	45.00	**95.38**
Hay-windrowed	99.37	98.51	99.57	99.04	**100.00**	**100**	98.73	**100.00**
Oats	5	38.50	11.50	63.50	16.50	**100**	48.00	**100.00**
Soybeans-no till	61.03	74.91	66.69	80.79	96.27	96.34	80.74	**99.23**
Soybeans-min till	74.46	84.51	80.23	87.66	**99.96**	99.91	90.41	99.93
Soybeans-clean till	68.96	82.20	72.98	84.98	98.50	**100**	82.85	**100.00**
Wheat	96.75	99.15	99.39	98.96	**100.00**	**100**	98.77	**100.00**
Woods	95.04	95.20	95.65	96.51	**100.00**	99.69	97.26	**100.00**
Bldg-grass-tree-drives	67.13	73.05	64.08	70.45	95.47	99.50	83.53	**99.89**
Stone-steel towers	69.26	70.32	70.42	77.05	99.58	98.63	98.63	**99.89**
OA	75.76	84.34	79.43	86.93	98.26	99.05	87.95	**99.75**
AA	63.66	77.72	67.15	82.44	91.51	98.47	83.47	**99.53**
k	72.22	82.09	76.38	85.06	98.02	98.92	86.36	**99.72**
Execution Time (seconds)	0.02	0.41	0.19	0.31	38.74	40.70	39.59	38.95

**Table 3:** Pavia University: overall, average, and individual class accuracy (in percent) and k statistic of different classification methods with 10% training samples. The best accuracy in each row is shown in bold.

Class	SMLR	KSMLR	LELM	NLELM	SMLR-LBP	KSMLR-LBP	NLELM-LBP	PROPOSED METHOD
Asphalt	72.27	89.43	85.27	88.82	98.62	**99.63**	99.49	**99.63**
Meadows	79.08	94.16	92.17	94.61	93.70	99.34	99.88	**99.83**
Gravel	71.99	85.08	78.06	87.41	99.14	99.64	99.92	**99.83**
Trees	94.90	97.92	97.38	98.16	99.27	**99.86**	98.54	99.64
Metal sheets	99.58	99.34	98.85	99.39	**100.00**	**100.00**	100.00	**100.00**
Bare soil	74.26	94.77	93.90	95.43	99.93	**100.00**	100.00	**100.00**
Bitumen	78.66	93.82	93.69	95.34	**100.00**	**100.00**	100.00	**100.00**
Bricks	73.37	87.52	90.05	90.94	99.93	99.63	99.85	**100.00**
Shadows	96.88	99.61	99.70	99.97	**99.89**	99.87	94.14	**99.89**
OA	78.78	93.00	91.23	93.94	96.93	99.59	99.62	**99.83**
AA	82.33	93.49	92.12	94.56	98.94	99.77	99.09	**99.87**
k	72.73	90.82	88.54	92.04	95.98	99.46	99.49	**99.78**
Execution Time (seconds)	0.19	4.40	0.48	3.83	**1193.7**	1237.1	5288.6	1201.2

**Table 4:** The classification results of the proposed method and other methods. The best accuracy in each row is shown in bold.

Datasets	Index	EMP-ELM	S-ELM	G-ELM	PROPOSED METHOD
Indian Pines data set with 10% training samples	OA	-	97.78	99.08	***99.75***
	AA	-	97.10	98.68	***99.53***
	k	-	97	98.95	***99.72***
Pavia University data set with 9% training samples	OA	99.65	-	-	***99.83***
	AA	99.60	-	-	***99.87***
	k	99.52	-	-	***99.78***
